# ‘No Sweet in Sex’: Perceptions of Condom Usefulness among Elderly Yoruba People in Ibadan Nigeria

**DOI:** 10.1007/s10823-018-9354-8

**Published:** 2018-08-20

**Authors:** Ojo Melvin Agunbiade, Dimeji Togunde

**Affiliations:** 1 Department of Sociology and Anthropology, Obafemi Awolowo University, Ile-Ife, Nigeria; 2 Department of Sociology, University of the Witwatersrand, Johannesburg, South Africa; 3 African Population and Health Research Center, Nairobi, Kenya; 4 International Studies Program, Spelman College, Atlanta, GA USA

**Keywords:** Condom, Unprotected sex, Sexual infections, Sexual pleasure, Older people, Nigeria

## Abstract

Emerging evidence has shown a gradual increase in sexually transmitted infections among elderly. This study explores the views of elderly Yoruba men and women (60+) on condoms use and its suitability against sexual infections. The research design was a sequential exploratory mixed method that consisted of vignettes based focus group discussion and a structured questionnaire. Twelve vignettes based Focus Group Discussion and a survey of 252 elderly Yoruba people (aged 60+) were carried out. The findings revealed limited awareness and experience with condoms. Few of the male (20.4%) and 2.8% of the female respondents felt condom use can prevent sexually transmitted infections. A marginally proportion of the females (29.2%) than the males (25.0%) perceived condom as more useful for younger people. Condom use as a preventive measure against sexual infections varies by gender and marital status (*p* = .000). Gender and marital status also had an influence on whether condom use could reduce sexual pleasures (*p* = 0.000). These findings offer strong support of the sexual pleasure hypothesis which is often invoked to explain attitude toward condom use in a variety of social and cultural contexts. Social marketing of condoms is urgently required to address misconceptions around condom use and encourage uptake among sexually active elderly people.

## Introduction

Condoms are useful protective measure against sexually transmitted infections (Holmes et al. 2004). Amongst other modern contraceptives, condoms can reduce the possibility of contracting or spreading sexual infections during penetrative sex (Fitch et al. 2002). Like any other modern contraception, there are psychosocial and cultural factors that hinder effective and adequate adoption of condoms as a preventive measure pleasures (Ellis et al. 2015). One of such beliefs include the normative conception of sexual pleasure as penetrative and that condoms use reduces such pleasures (John et al. 2015).

Paradoxically, the perception that condom use can reduce sexual pleasures competes with the consideration to prevent sexual infections and transmission to partners during intercourse (Ellis et al. 2015; John et al. 2015; Randolph et al. 2007). The perception that condom affects sexual pleasures exist among different social categories and there are indications that the impression can be modified through different behavioural interventions. In this regard, some scholars have called for efforts to improve the effectiveness of condoms to prevent sexual infections and also reduce its effects on sexual pleasures (John et al. 2015; Khan et al. 2005). As a result, promoters of condoms and other stakeholders have deployed attention to different strategies that can scale up condom use among young and middle-aged adults (Sweat et al. 2012). Unfortunately, these efforts have targeted young and middle-aged adults at the expense of older adults. Partly, the skewness might be associated with dominance of epidemiological evidence on sexual infections in reproductive years. There is also the perception that older people are mostly fragile and prone to age-related health challenges, which make them to be less interested in sexual activities.

Emerging evidence from different cultural settings have shown otherwise. A multi-site study that covered Spain and the United States, for instance, reported that risky sexual practices such as multiple sexual partners and dislike for condom also exist among some older adults (Foster et al. 2012; Lindau and Gavrilova 2010; Lindau et al. 2007). Similar findings have been observed among older people in Malawi (Freeman and Coast 2014) and Uganda (Negin et al. 2016). A study among older people in China also revealed some older Chinese males have multiple sexual partners and also vulnerable to contracting sexual infections (Zhou et al. 2014b). In Nigeria, some studies have also reported intergenerational dating, multiple sexual relations (Ojebode et al. 2010), extra marital affairs, and unprotected sex between young people and older adults (Okonkwo 2013). With the global and regional increase in ageing population, it is worrisome to sustain the unempirically informed impression that a small propotion of older adults are contracting sexual infections and that their sexual health needs is insignificant compare to other health needs in later life. The gaps in the gerontological literature on sexual health in old age constitutes some levels of risk, especially given the skewness of research and policy to sexual and reproductive health issues in the sub-Saharan Africa (Aboderin 2014). The availability of contextualised evidence on sexual health needs and practices in old age can provide directions on how to address the challenges and improve ageing experience and healthy ageing within a given social setting. Such body of evidence could also improve existing discourse on the diversity and heterogeneity of sexual health needs in old age (Bell 2016).

In bridging the gaps for contextualised evidence, this paper investigates condom awareness, reasons for use or non-use and its perceived suitability against sexually transmitted infections among older Yoruba men and women (60 years and over) in Ibadan, Nigeria. All social actors have the potentials to perceive sexual risks differently and the practices that can promote or mar their sexual health and well-being across their life course. It is critical to understand this social reality among elderly people as this will improve the body of knowledge that is relevant to addressing the unmet need for sexual health care services in later life. Thus, we argue that a contextualised understanding will provide insights to the intersections between normative sexual beliefs, values and practices that can compromise or promote sexual health in old age. We aim at this goal by exploring the perceived risks of use and non-use of condoms and the alternative practices that older people can or do deploy to reduce their vulnerability or otherwise to sexual infections along the life course.

## Gender and Variations in Sexual Risks in Old Age

The assumption that older people and their sexual health needs are homogenous contradicts empirical evidence. The same also applies to the stereotyped perceptions of older people as asexual, homogenous, fragile and prone to age-related health challenges, which make them less interested in sexual activities (Gott and Hinchliff 2003; Moore 2010). There are risky sexual practices among older people. The existence and indulgence in such practices, however, varies within and across gender (Corona et al. 2010; Foster et al. 2012; Lee et al. 2016). The variations are also connected with health-related factors and cultural worldviews around bodily changes and sexuality (Lee et al. 2016). Across the life course, health related factors intersect with other contextual variables such as the dominant and marginal beliefs and values around sexual intercourse to influence the premium, dispositions and indulgence in sexual activities among older adults in a given social setting (Agunbiade and Ayotunde 2012; Freeman and Coast 2014; Zhou et al. 2014a).

In expanding contextual evidence on gendered variations, a postal survey conducted in the United Kingdom, Gott (2004) found that 7% of older people engaged in activities that could predispose them to contract Sexually Transmitted Infections (STI). The results demonstrated gender differences: more men than women were engaged in risky sexual practices. It is tempting to think that indulgence in sexual risks might be associated with knowledge and ignorance of the possible implications. Sometimes, it might be true, however, it is also possible to rationalise the possibility of contracting an infection even while indulging in sexual risks. Among the older Chinese men in Zou et al. (2014a) study, some older men were aware of the implications of engaging in sexual risks, however, they still rated low the likelihood of coming down with a sexual infection. A possible explanation could be found in the social construction of risk as common among younger men than older men. A situation that has been reinforced in diverse ways through gendered roles and normative expectations. Across the life course, men and women are socialised into different sexual roles and responsibilities, which impact on their sexual behaviour and health outcomes (Varga 2003). As individuals grow older, the particularity in their sexual behaviours and practices are sometimes lost to the dominant and marginal stereotypes and misconceptions around sexuality in old age (Freeman and Coast 2014; Gott 2004; Zhou et al. 2014a). The consequences partly include non-availability of sexual healthcare services for older people and the view that there are other health issues that are of critical value to well-being and health ageing experiences (Agunbiade 2016; Freeman and Coast 2014). Other ripple effects include poor sexual health outcomes and dominance of subjectively assessed practices as effective measures of minimising negative sexual health outcomes in old age (Agunbiade 2013; Zhou et al. 2014a).

To understand the differences and similarities in sexual behaviours within age cohorts, Calasanti (2010) argued that a contextualised understanding of the perceptions of sexual risks between and across gender could be fruitful. Calasanti’s (2010) view is consistent with existing findings portraying gendered variations in sexual risks and practices (Corona et al. 2010) and age cohort differences in the formation of sexual attitudes and practices (Mercer et al. 2013). In this regard, we found the age-graded perspective to sexuality useful. The age-graded approach as described González (2007) depicts how social actors are indoctrinated into structural arrangements that recognises and places certain social expectations about age in the demarcation of social rights, duties, and expectations including sexual activities. Across the life course, individuals as social actors are socialised into what they can do or not do with their bodies within time and space (Calasanti 2010). However, without relinquishing individual agencies, the right to exercise personal choices are located within normative relations with possible influence on decisions and actions. In societal wisdom, the presumptions include the subjective assessments that the freedom to excercise certain priviledges including sexual rights might be consequential on personal well-being and by extension that of others their social settings. However, the reality is that these normative expectations are sometimes challenged as individual agencies are expressed through diverse sexual orientations as events and challenges emerges in life their life span (Simon 1996). 

In patriarchal settings, including the Yoruba culture where this study was conducted, polygyny is socially practiced with social laxity and social expectations that men’s sexuality is superior to that of their female counterparts. Through the life course the social expectations around male sexuality are transmitted in diverse forms and structures including their right to initiate sexual desires as they grow older. Similar expectations are unexpected of females, even when they increase in age. Restrictions and repression of sexual desires are encouraged including the need to take passive roles in sexual initiations.

Despite the existence of evidence in support of diversities in sexual desires and expressions at various stages in life, the likelihood of denying individuals and certain social categories their private rights is in late-adulthood. This contrasts mid-adulthood, where fertility attracts high premium and is one of the excusable reasons for indulging in sexual intercourse. Shortly after mid-adulthood, there is a gendered differential. Females, for instance, are expected to disengage or repress their sexual desires, while their males counterparts are somehow motivated to continue. The situation is not universal, there are cultural variations in the restrictions on sexual desires and expressions in post-reproductive period. Among the Yoruba speaking people where this research was conducted such restriction becomes vivid and enforced in diverse ways for women as they grow older. In contrast, voluntary disengagement is expected of older Yoruba men as they can initiate new sexual relations. In reality, absolute compliance is unrealistic as both males and females could deviate from these restrictions and societal expectations. For factors such as sexual history, experiences and perhaps hormonal variations, the likelihood to develop an impression that sexual activities are somewhat beneficial in later life can occur. This is hinged on the view that individuals have the capacity to deploy their agencies differently and act according to their private beliefs when there are opportunities for them to express their sexual desires (Agunbiade 2016). Within this social framing, we explore the possible varied meanings older men and women can attach to condom use, as a protective measure against sexual infections and the possible influence on sexual pleasures. In this regard, this paper seeks to contribute to the theoretical and empirical body of knowledge on the intersections between sexual pleasures and normative values around sexual-risks in old age.

## Methods of Data Collection and Analysis

The findings presented in this paper are part of a larger study on sexuality, ageing and help-seeking behaviour among elderly Yoruba People in Southwest Nigeria. The larger project was guided by a sequential exploratory mixed method design (Creswell and Plano Clark 2011; Hesse-Biber 2010, p. 3), which consisted of a larger qualitative component in the form of a vignette based focused group and semi-structured interviews. The qualitative data collection took place started in April and ended in October 2014. The survey was conducted between February and March 2015. The survey is the lesser component of the study design, which involved the use of a structured questionnaire that was developed from the qualitative findings. In this paper, attention is restricted to the evidence generated from both sources in relation to condom use, experiences and perceived suitability of condoms as preventive strategy against sexual infections.

The study population consisted of older Yoruba men and women aged 60+ who are currently residing in Urban Ibadan in South west Nigeria. As such, the study was conducted in 6 purposively selected communities in Ibadan North and Southeast Local Government areas in Oyo State Nigeria. These communities are in the inner core of Ibadan. Residential developments in the southwest Nigeria are broadly categorised into high, medium and low-density areas (Jiboye 2014). On the average, high-density residential areas in Ibadan have fractions of modern health facilities and a large proportion of traditional health practitioners (Lawal et al. 2015). The house-types in the inner core areas are roomy and often occupied by family members with limited or absence of rooms for rent. Furthermore, the buildings are old and spatially netted together in a way that two buildings share the same fence. These, among other factors, reflect the socioeconomic characteristics of the residents (Jiboye 2014), and could increase the frequency of interaction and co-residency among the populace. Indigenes and early settlers are mostly found in the inner core areas.

A few non-indigenes reside in these areas due to limited options of securing rented apartments despite the low cost of rents within the vicinity. Nonetheless, the spatial arrangement creates wide network of relations and interactions. The reasons are not surprising; social relations in inner core areas are predominantly informal, with a high tendency to interfere in private matters and lives of others.

### Recruitment Approach in the Qualitative Phase

A gradual selection approach was also adopted to gain entrance into the community and develop a good rapport with the research participants. After the initial identification of the six communities, the mobilisation officers at the Local Government Areas where these communities are located then provided links to the community leaders. The community leaders acted as gatekeepers and facilitated the recruitment of participants for the FGDs. All the community leaders were briefed on the research aims and objectives. They were briefed on their rights to voluntary participation and withdrawal from the research. They were also encouraged to ask questions concerning the research. The inclusion criteria for the research were also provided and the rationale was explained. In each of the study locations, two community leaders were selected. Each community leader, a male and a female, assisted in recruiting participants of their gender. Each community leader was assisted by a fieldworker in the recruitment of potential FGD participant. The involvement of community leaders in the recruitment of participants contributed to the response rate that was achieved at the three stages of data collection in the larger research, out of which the data from this paper was drawn.

A stratified purposive sampling approach guided the recruitment of participants. This approach involves the segregation of participants into sub-groups based on relative homogeneity of characteristics. In stratified sampling design, the social category of research interest is divided into strata and then a small number of cases is purposively selected within each stratum for in-depth study (Teddlie and Yu 2007). In this research, participants were classified and recruited based on gender (male/female), age categories (60–69, 70–79 and 80 years and above) and ethnicity (Yoruba).

Specifically, 12 FGDs with vignettes were conducted as a function of gender among the elderly Yoruba men and women in three age categories (60–69 years, 70–79 years, and 80 years above). Two variants of qualitative vignettes were constructed and validated using a team of selected nine experts in sexual health research. Both vignettes differ based on gender of the character and normative gendered practices in heterosexual marriage among the Yoruba people. The qualitative vignettes were then used to initiate focused discussions on normative views on sexual practices, possible deviations as experienced by the research participants or their peers and their perceptions on the existence and possibility of contracting a sexual infection in old age.

Across the three age categories, 107 elderly Yoruba men and women featured in the FGDs. In consonance with the literature (Krueger and Casey 2009), an average of 9 males participated in the 6 FGDs held with elderly men while an average of 8 participants featured in the 6 FGDs with women. On the average, the FGD session lasted for 92.1 min (1 h, 33 min).

## Data Analysis

### Qualitative Phase

All the 12 vignette-based FGD were in Yoruba Language and then translated into the English Language. Two experts in Yoruba and English languages conducted a back-translation of the transcripts for clarity in the representation of the participants’ views (Chen and Boore 2010). Areas of divergence between the two language experts were resolved before coding the transcripts. The thematic approach suggested by Boyatzis (1998) was adopted in the analyzing the data at two levels. The first stage was a quick analysis of the data based on the themes that guided the data collection. At this point, salient themes and subthemes emerged for the second stage of the analysis. In the second phase of the analysis, all the translated transcripts were edited and transferred into Nvivo 10 for further analysis. At this stage, further coding was conducted, with themes and sub-themes that explained the research questions being identified. At all levels of the analysis, both deductive and inductive coding approaches were used to analyse the data until a saturation level was achieved.

### The Quantitative Phase

The different views and consensus that are inherent in the salient themes were structured into a questionnaire, pre-tested and interviewer administered among 252 elderly Yoruba people(60+). The aim here was to understand the commonality and wide spread nature of the convergent and divergent views that emerged from the qualitative findings around the use of condoms as a preventive measure. Weights were assigned to selected items and further computations were carried out. Simple frequency was done first, and subsequently, a descriptive statistical technique (chi-square) was computed to determine the level of association among the variables of interest. At each point of the data presentation and analysis, relevant quantitative results were used to complement the qualitative findings.

## Ethical Consideration

As expected in any data collection that involves human subjects and sensitive issues, we have followed all ethical considerations by obtaining participants’ consent to ascertain that participation is voluntary. In addition, permission was sought from the local community leaders along with prior institutional ethical approvals from the Research Ethics Committees at the University of the Witwatersrand, South Africa and the Obafemi Awolowo University, Ile-Ife, Nigeria.

## Findings

The qualitative findings has two salient themes. These themes are: awareness and perceived usefulness of condoms in old age and experiences associated with condom use. The results from the survey complemented the qualitative findings on awareness, perceptions of condom usefulness and experiences with the use of condoms in old age.

## Profile of the Focus Group Participants

An average of nine males participated in the six FGDs held with elderly men while an average of eight participants featured in the six FGDs with women. Polygynous marriage was predominant among the FGD participants. Less than a quarter (23) of the participants was in monogamous marriage. A higher proportion of the participants were Muslims (71). Only 24 participants were Christians, and 3 were from the traditional Yoruba religion.

## Socio-Demographics of the Survey Respondents

A slightly higher proportion of the females (57.14%) than the males (42.86%) participated in the survey (please see Table 1). The gender differential in the percentage of the survey respondents is consistent with the literature. More females than males survive into old age owing to the interaction of biological, psychological and social factors. Close to two-thirds of the survey respondents were in the age bracket 60–69 years. Similarly, over 50% of the respondents were married. The majority (68.3%) of the total respondents were Muslims. Islam is a dominant religion in Ibadan, along with Christianity. Each of the study sites had conspicuous religious worship centres that were potentially accessible to members of both faiths. A few such places of worship existed for traditional religions. In other words, both Islam and Christianity are somewhat accessible to new followers. This may be associated more with wider socio-political factors than with differences in doctrines and practices. Formal education was low among the respondents, as more than one-third had no formal education. Less than 35% had primary education, which cut across the three age categories and gender.

**Table 1:** Socio-demographic characteristics of the survey respondents

	Freq.	Percent
Gender		
Female	144	57.14
Male	108	42.86
Age Categories		
60–69	137	54.4
70–79	75	29.8
80 years and above	40	15.9
Marital Status		
Married	151	59.9
Widowed/Divorced	101	40.1
Religious Affiliations		
Christianity	80	31.7
Islam	172	68.3
Educational level		
No formal	139	55.2
Primary	78	31.0
Secondary and above	35	13.9

Most (84.92%) of the respondents engaged in various economic activities to earn a living. A similar proportion (75.79%) relied on their children for financial support. This is analogous to the characteristics of the FGD participants. Informal support for the elderly is diminishing, as more elderly people still work to earn a living. As stated earlier, the lack of a social security system to cater for the financial needs of old people and an inadequate level of familial and cultural responsibilities for older people may be compounding the situation (Adeniyi-Ogunyankin 2012).

## Awareness and Perceived Usefulness of Condoms in Old Age

The awareness older people have about condoms seems high among the male respondents in this study. This is occurring despite the marginalization of elderly people (60+) in the social marketing of condoms in Nigeria (Sunmola 2005). Existing empirical evidence on condom use shows that awareness of and association of condom use with the prevention of infections do not imply the proper and efficient use of a condom. Other factors, such as stigma, or shame, and the feelings that the use of condom can reduce sexual pleasures can influence older people’s disposition to condom use (King and Olaseha 2012a; Ludwig-Barron et al. 2014).

Regarding awareness, the male FGD participants claimed that they had seen and were more aware of male condoms than were the female participants. A few of the females also claimed their awareness of the male condoms, but none had seen a female condom before. For sexually active old men, the use of a condom was greeted with mixed feelings. The majority (39) of the elderly men acknowledged that the use of condom during penetrative intercourse can reduce the contraction of some sexual infections. However, the bursting of condoms and the possible reduction in sexual pleasures discouraged them from using them except on rare occasions. Largely, some of the sexually active elderly men conceived condom as useful for younger people to reduce unintended pregnancy and sexually transmitted infections. The following extracts exemplify their views on the usefulness of condoms:

It can prevent unintended pregnancy among young people, especially those in schools. [FGD with women aged 70–79 years, Sango Community].

If a condom is used, it will avoid the spread of disease/infections. [FGD with men aged 60–69 years, Bodija Community].

By using a condom, we know it is for prevention, but no enjoyment can be derived from it. It is a waste of energy. [FGD with men aged 60–69 years, Bodija Community].

The few male participants (5) who shared personal experiences with condom use emphasised the negative influence of condom on pleasurable penetrative sex. Three among the elderly men between 60 and 79 years of age claimed that they had used condoms at least once within the last few months. Within this category of men with recent sexual activities, condom use was rare and described as being less pleasurable. As shared by one of these men, the use of condom symbolises waste of energy except when a man perceives that there are possibilities of contracting a sexual infection. In his words:

I can use it if a woman insists and I sensed that I can contract a sexual infection. (A 69-year-old participant, FGD with men aged 60–69 years, Bodija Community].

In summary, the qualitative findings revealed a wide acceptance and belief that some sexual infections are preventable, but such views were expressed more among men. In addition, there was inadequate faith in the use of condoms among the men that reported recent sexual activities. For instance, the excerpts from the above male FGD participants portrayed a negative effect of condom use on sexual pleasure and satisfaction. Hence, it became necessary to conduct a survey to understand a wider pattern and views of elderly people to condom use as a preventive strategy against sexual infections.

In Table 2, the survey results showed few of the male (20.4%) and female (2.8%) respondents felt condom use can prevent sexually transmitted infections. A marginally proportion of the females (29.2%) than the males (25.0%) perceived condom as more useful for younger people. In terms of possible predicting factors, gender and marital status were found statistically significant. At 0.05 level of test, gender and marital status influenced the view that condom use can prevent sexually transmitted infections (*p* = 0.000). However, only gender was significant on whether condom could reduce sexual pleasures (*p* = 0.000) (See Table 2 for additional results).

**Table 2:** Selected demographic factors and some views on condom use in old age

	Gender		Age			Marital status	
Statements on condom use in old age	Women (*n* = 144)	Men (*n* = 108)	60–69 (*n* = 137)	70–79 (*n* = 75)	80+ (*n* = 40)	Married (*n* = 151)	Widowed/Divorced (*n* = 101)
Condom use can prevent sexually transmitted infections Yes	11.1	42.6	27.7	20	22.5	33.1	11.9
Sig. Test	χ^2^ = 32.972; d.f. = 1; *p* = 0.000		χ^2^ = 1.678; d.f. = 2 *p* = 0.432			χ^2^ = 14.707; d.f. = 1; *p* = 0.000	
I can use condoms to prevent sexually transmitted infections Yes	2.8	20.4	11.7	8.0	10	14.6	4.0
Sig. Test	χ^2^ = 20.643; d.f. = 1; *p* = 0.000		χ^2^ = 0.714; d.f. = 2 *p* = 0.700			χ^2^ = 7.362; d.f. = 1; *p* = 0.007	
The use of condoms can reduce sexual pleasure Yes	6.9	32.4	19	17.3	15	25.2	6.9
Sig. Test	χ^2^ = 27.279; d.f. = 1 *p* = 0.000		χ^2^ = 0.354; d.f. = 2 *p* = 0.838			χ^2^ = 13.719; d.f. = 1; *p* = 0.000	
Younger people will find condoms more useful than elderly people Yes	29.2	25.0	73.7	69.3	75	74.8	69.3
Sig. Test	χ^2^ = 0.539; d.f. = 1; *p* = 0.463		χ^2^ = 0.605; d.f. = 2; *p* = 0.739			χ^2^ = 0.930; d.f. = 1; *p* = 0.335	

From the perspectives of the male participants, condom use can prevent sexually transmitted infections. Among older people, engagement in extramarital affairs and unprotected sex with multiple partners can lead to an infection. For older people who engage in multiple sexual relations, there are chances of contracting gonorrhoea, syphilis, HIV and *magun*. For the participants who shared this opinion, gonorrhoea, syphilis and magun are common. Some of the respondents also mentioned that they knew elderly people have contracted one of these infections in their communities apart from HIV. For both the male and female participants, the chances of contracting gonorrhoea or magun was higher than that of syphilis. Their explanation was hinged on some normative beliefs that unfaithfulness and infidelity are common among young and old and therefore provides opportunities for sexually active people to contract an infection.

In terms of prevention, condom was considered useful in reducing the possibility of contracting gonorrhoea, syphilis and HIV. For magun, prevention with the use of condom was described as ineffective. Their explanation rests on the aetiology of magun and its symptoms as a folk sexually transmitted condition. In the Yoruba belief system, there are different types of *magun*, and each one has its remedy, which also differs from one medical practitioner to other (Ogunsakin-Fabarebo 1998). The make-up and antidotes to each *magun* are different from one medical practitioner to another despite the similarities in symptoms and outcomes. In the Yoruba belief system, *magun*-related deaths are ways of punishing sexual immoralities in marriage (Alaba 2004; Esonwanne 2008). *Magun* is a magical substance placed on a woman for her sexual partner to contract (Alaba 2004). It can be put by any man on any woman to eliminate her sexual partner or as a form of warning to the woman:

A special condition that was mentioned among the male

Once *magun* is placed on a woman and she knows it, she goes to the traditional doctor to give her an antidote. If she is not aware, and had sexual intercourse with any man, it is instant death for the man. If she doesn’t receive any antidote and no sexual intercourse within a given period, death still remains the ultimate for a woman with *magun*. [FGD with women aged, 70–79 years, Sango Community].

…such thing is still common among Ikire women because they do not stay in their husbands’ homes. Their men do not like people to have affairs with their wives. Hence, they always put *magun* on their wives; one should be very careful unless one has a preventive measure for it. Ikire women hardly stay in their husbands’ homes and are promiscuous, so their husbands often place *magun* on them. [FGD with men aged 60–69 years, Bodija Community].

If *magun* should be put on a woman and that man too is a good adulterer, there are antidotes that he will use to draw it up (render it impotent) until he finishes. Some have immunized themselves against it; these are purely local and not Western drugs as such. [FGD with women aged 70–79 years, Sango Community].

Depending on the contexts, religious beliefs and social position or status, a man can decide to place *magun* on his wife, even when there are unconfirmed facts, to eliminate her supposed concubine or sexual partner. The social expectation is that within a short period, any man who has sex with such a woman would die immediately or within few days. Unlike the biomedical prognosis of disease, *magun* and associated symptoms defy empirical evidence and hardly provide any allowance for recovery (Agunbiade and Ayotunde 2012). The antidote to *magun* is avoidance of sexual relations with the woman-carrier or possession of a remedy that would seize the power of *magun* during intercourse (Aderinto 2012; Moloye 1992).

Only certain individuals possess the antidote and the knowledge of manipulating the power of *magun* before and during intercourse with a *magun-*carrying woman*.* This procedure is only temporal, a permanent removal and neutralisation of the *magun* effect often resides with the person that implants it. The woman carrying the *magun* is still capable of causing sudden death for whoever sleeps with her. The argument of the participants is that, once *magun* is placed on a woman, if not removed within a stipulated period, death is inevitable for the carrier. If a woman with *magun* also sleeps with her husband who lacks the knowledge and power to curb *magun,* his death is also inevitable. This explains the practice of shifting the potency of *magun* in an infected woman for sexual intercourse without the power to remove the *magun* completely except by the person who planted the *magun*. In principle, men that engage habitually in extramarital relations must protect themselves against sexually transmitted infections, particularly *magun*, as indicated in this excerpt:

If one is covetous because women are dangerous in terms of infections, one should have prevention against such infection. For instance, there is *magun* – a form of infection that kills immediately. Any man that has a desire for extramarital affairs must be prepared so that if he encountered any woman with *magun*, he would survive it. [FGD with men aged 60–69 years, Bodija Community].

Based on the aetiology and symptoms associated with *magun*, the participants commented that only traditional medical means can effectively eliminate the effect of *magun* on an adulterer. The use of biomedical means, such as condoms and vaccines cannot remove the effects of *magun*:

Condom cannot prevent an individual from contracting *magun.* The only prevention is for a man not to mount or climb a woman that is infected with *magun.* [FGD with men aged 80 years and above, Oniyere Community].

An individual that has the knowledge and the antidote can have intercourse with a woman that has *magun.* This requires the use of charm to render it feeble for the man during intercourse. He will have enjoyable sex with the woman conveniently without any harm since he has the antidote. However, if another man tries the same without using the right antidote, death is certain. [FGD with men aged 80 years and above, Oniyere Community].

The logic behind *magun* is inexplicable. It is magical in its operation. The fear that some married men, including the elderly ones, engage in extramarital relations clouded the discussion among the female participants. As a form of protest, the female participants declared their support for women who would avoid sexual intercourse, especially in menopausal age to curtail being infected. Having worked and sacrificed their individual agencies during the reproductive period, the post-reproductive stage demands vigilance, as all efforts must be in place to avoid being beaten twice. Similarly, some of the female participants also approved of the use of *magun* as a practice to enforce fidelity and punish marital infidelity.

## Experiences Associated with Condom Use

With long years of sexual activities and experiences, elderly men might experience less pleasure using condoms during sex. From two of the FGDs, some of he male FGD participants described condom use during sex as being less satisfying and undesirable:

‘No sweet in using condom’ and that is why some women will never agree with their husbands or partners to use a condom with them. [FGD with men aged 60–69 years, Bodija Community].

The use of a condom decreases the pleasure, and it could break when used. The fear of it breaking during intercourse and the possibility of not having adequate pleasure discourage people. [FGD with men aged 70–79 years, Inalende Community].

Concerning the issue of displeasure in the use of male condoms, different experiences and practices were reported among three male participants. In the FGD with older males aged 60 to 69 years, a male participant described how he once circumvented the proper use of condoms for pleasure. In his opinion, he did a subjective assessment of his sexual partner and concluded that she was free of any sexual infection. In his narrative, the desire for pleasure would sometimes prevail over the possibility of contracting an infection. He narrated a recent experience that demonstrated such a dilemma:

Once I’m sure, and I want the pleasure, I will use a trick to cut the condom tip without the woman’s knowledge. Through this, I will have it flesh-to-flesh with her. However, if you want to prevent it, you do not need to make holes on it. [A 69-year-old participant, FGD with men aged 60–69 years, Bodija Community].

The possibility of circumventing the use of condom by some men was also mentioned by a female FGD participant who eavesdropped on a conversation at the State Hospital between a nurse and a nursing mother that had an unintended pregnancy. In her account, perceived contraceptive side effects prevented the nursing mother from using a contraceptive. Therefore, her husband agreed to the use of condom during intercourse. Unfortunately, the husband circumvented his wife by pretending to use a condom during intercourse. This only became known to the woman after she became pregnant despite breastfeeding a 9-month-old baby. Below is the extract:

The condom may prevent, but some men are wicked. My experience on the day I took my child to Adeoyo (a state hospital), a woman with a 3-month old baby was crying that the nurse should help her. She was asked to call her hubby, and when he came, he said that the condom is not compatible with his body that is why he would cut the condom. Everybody blamed him that is he is wicked, that he wanted to kill the new baby. That is why some women may try to avoid their man or refuse to use a condom if she does not wish to be pregnant. [FGD with women aged 60–69 years, Bodija Community].

Sexual engagements and pleasures in old age depend on both relational and contextual factors, which might affect both genders differently (Ménard et al. 2015). In the FGDs and some of the interviews, factors within relationships and contexts were brought to the fore as the participants define and interpret outcomes from sexual engagements. In relation to condom use, agreement between partners and equal participation was conceived as a necessity for actual use of a condom during intercourse. In the absence of consensus, the possible risks of unprotected sex were swept under the carpet as demonstrated in the experiences of some of the male participants. For this category of males, displeasure concerning the use of condoms based on perceived reduction of sexual pleasure ranked higher above possible contraction of sexual infection. Although, the possibility of contracting a sexual infection was given credence, but as earlier stated, experienced polygynous men are to guide against actual contraction of an infection with the use of traditional medicine. Once such preparation is guaranteed, pleasure from penetrative sex was considered vital. The emphasis on pleasure could be observed in the narratives and rationale as exemplified in some of the following extracts:

There is no enjoyment in using condoms. I will never use it. Rather than using it, to avoid contracting an infection, then I will just romance such a woman and let her go until I get another woman that may be free of infection and also agrees with flesh-to-flesh. [A 63-year-old participant, FGD with men at Bodija Community].

The body contact brings out enjoyment in sexual intercourse. The only enjoyment one derives is when you are about to discharge; apart from that, there is no enjoyment in it. [60-year-old participant, FGD with men at Bodija Community].

When we engage in sex, we desire pleasure, and it comes from skin-to-skin sex (*chorus response*). Condom use makes it different; the direct experience becomes threatened. [FGD with men aged 70–79 years, Inalende Community].

To me, I dislike the use of a condom. What’s the relevance of using it when you will ejaculate and again use your hand to remove what you have released? It is better not to have sex when your semen is wasted or not useful for a woman’s body. It does not bring any satisfaction, and it is better not to use it since it reduces the pleasure. [81-year-old participant, FGD with men at Inalende Community].

Judging from the extracts above, it will require more efforts to optimise the protective benefits of condom use across different social categories. It is possible that the use of condoms during penetrative sex could have negative or positive effects on sexual sensations and pleasure. At some points, the male FGD participants interpreted derived sexual pleasure similar in orientation and expectations to those of their partners.

## Discussion

In this paper, we focused our attention on condom awareness, use and experiences among urban dwelling elderly Yoruba people aged 60 years and above in Ibadan, Southwest Nigeria. Special attention was given to their notions of heterosexual activities and penetrative vaginal sex and how these interpretations could influence their dispositions towards condom use. This study is the first to investigate the perceptions of and condom use experiences of urban dwelling Elderly Yoruba people (60+). The study employed a sequential exploratory mixed method design, which also served as a methodological contribution to the few existing studies on sexuality in Old age within the Nigerian context (Adeoti et al. 2015; Agunbiade 2013; Agunbiade and Ayotunde 2012; King and Olaseha 2012b) and in some other contexts within and outside Africa (Freeman and Coast 2014; Van Der Geest 2001; Yan et al. 2011). The adoption of an exploratory sequential mixed methods approach provided a culturally sensitive and unique approach to assess sexuality and existing practices among elderly people. It also demonstrated the possibilities of exploring the intersection between ageing and sexuality from the perspectives and lived experiences of elderly people. The dearth of mixed methods design in gerontological literature has somehow contributed to the limited voice of older people and the existence of diverse insights in the literature (Fredriksen-Goldsen and Muraco 2010).

The findings supports some of the earlier studies showing inadequate awareness and low perceptions of condoms usefulness among elderly people in Nigeria (King and Olaseha 2012b) and Malawi (Freeman and Coast 2014). In part, the inadequate awareness and low perception might reflect the sustenance of cultural premium on heterosexual relations and procreation across the life course. With the neglect of older adults in the social marketing of condoms, it might be easier for older adults to pay less attention to public campaigns and initiatives that are targeted at promoting sexual health. Furthermore, the social expectation that elderly people should retire from sexual activities at a point in their life course can have negative influence on their sexual health and dispositions towards condom use. While the tendency to tactically omits elderly people from the sexual fields prevails, their vulnerability to sexual infections due to risky sexual practices and misconceptions is not guaranteed.

The absence of any significant relationship between age and the response to statements on condom use in the survey phase serves as a caution for us to not conclude about the applicability of the age-graded framework in this study. Instead, the findings lend much credence to the sexual pleasure hypothesis as the participants recognize the use of condom although they also thought that it can reduce sexual pleasures. This view, however, was shared more among men than women and more among the currently married than those widowed or divorced. The variations and particularity of values and relevance attached to condom use is consistent with existing findings that elderly people have sexual desires, express these in diverse ways and engage in sexual practices based on their capabilities (DeLamater 2012; Gott and Hinchliff 2003). It is noteworthy that the disparities and particularised capabilities for sexual practices within and across genders are hardly separable from dominant socio-cultural beliefs and practices associated with the kind of age-graded sexualities in a given context (Arnfred 2004; González 2007).

There are important implications from the findings. For instance, the overt emphasis on penetrative vaginal sex and cultural practices that could aid sexual pleasures can undermine sexual health in old age. As such, we support Wellings et al. (2006) position that there is need for more studies investigating the dynamics and context of involvement in unprotected risky sexual practices in later life. Partly, such studies could be useful in understanding the vulnerability of elderly people to sexually transmitted infections and the possible ways through which unmet need for post-reproductive sexual healthcare services can be addressed in a given cultural setting. Within the study context, earlier studies have shown that the existence of high premium on penetrative sex and the use of traditional aphrodisiacs among older men in polygynous marriages (Agunbiade 2013; Agunbiade and Ayotunde 2012). The expectation that the use of aphrodisiacs enhances sexual pleasures has a long history and exists within the biomedical and African traditional medical systems. This commodifies sexual pleasures and a reinforcement of the notion that the use of condoms reduces sexual pleasure. This utilitarian view was more widely shared among the male than female participants in this study.

From the findings, there are indications that sexual subjectivity is fluid and gendered as the participants attached different meanings and expectations to sexual activities and condon use experiences. This supports Fileborn et al. (2015) findings among older women in Australia where some of the participants narrated different expectations and outcomes from each sexual engagement with their partners at various periods. In this research, patriarchy and heterosexual normativity might be creating a false sense around males’ capabilities to determine sexual satisfaction and pleasure of their sex partners. For instance, wearing a sensational rubber during intercourse might produce different sexual feelings and pleasure for both partners even in a sexual episode. As earlier argued by Fileborn et al. (2015) and Ménard et al. (2015), experiences of sexual pleasure and satisfaction are fluid and dynamic for individuals and couples across the life course. In view of these findings, it is useful to situate the views and experiences of the elderly in this study within a larger body of evidence in gerontological literature.

There are limitations from this research. The study was carried out among older Yoruba people within a relatively homogenous social setting. A similar study among the various ethnic groups in Nigeria might show some variations. The reasons are not far fetched since age-graded sexual beliefs and practices abound across cultures (González 2007). Our speculation is that a study of this nature in a larger sample of older adults from within and outside the Yoruba ethnic group might produce some slightly different findings. As shown in this study, older people are relatively heterogeneous in their sexual history, experiences and practices. As such, these variations are also expected in their acceptance and conformity or compliance to the social expectation that sexual activities are more appropriate at a certain age in life and more useful for procreation purposes. Beyond the differences in sexual histories and experiences, gender also counts in the dispositions of older adults towards their sexual health (Calasanti 2010). For biological and cultural reasons, women within the study settings are socially expected to retire early from sexual activities especially in menopausal stages of their lives (Agunbiade 2016). Despite the limitations of this research, the findings reiterate the existence of individual agencies and the capability to engage in varied sexual activities within sexual fields across the life course (Green 2008). From an age-graded lens, we argue that by disapproving sexual expressions and desires in old age, elderly people are motivated to suppress or relinquish their sexual rights, but become somewhat vulnerable to unhealthy sexual practices. An investigation on awareness of sexually transmitted infections, cultural beliefs around aetiology and treatment outcomes can also provide contextual evidence on how to scale up uptake of condoms among older adults that are sexually active. The findings therefore draw our attention to some urgent policy gaps which are highlighted in the concluding part of this paper.

## Conclusion

Post-reproductive sexual health needs of the elderly people are growing, yet there is absence of concrete efforts to meet these needs and promote their sexual health. The social marketing of condoms- a relatively potent strategy of preventing sexual infections is tailored towards young and middle-aged people. This is having unintended consequences for elderly people. Currently, the awareness is low and there are misconceptions around the usefulness or irrelevance of condoms to elderly people. These perceptions are also connected with prevailing age-graded constructions of sexuality and pleasure. With the neglect and partial attention to post-reproductive sexual health needs, vulnerability to sexual infections may be on the increase among elderly people in the study setting. Social marketing that focuses on promoting awareness and use of condoms is urgently needed among elderly heterosexual men in Nigeria. Avenues for voluntary testing for sexually transmitted infections can aid early detection and protection of sexually active older people and their sexual partners from infections.

## Policy Implications

There is an urgent need for policies that will cater for the post-reproductive sexual health desires of the elderly in Nigeria. Such policies should include the provision and availability of post-reproductive healthcare services. There is need for public enlightenment and sex education on safe sexual practices among old people. Such intervention might be useful in reducing the misconceptions around condoms among sexually active elderly men.
